# Editorial

**DOI:** 10.34763/jmotherandchild.20212504.edit.2021_25_04

**Published:** 2022-09-02

**Authors:** Aleksandra Jezela-Stanek

**Affiliations:** 1 Department of Genetics and Clinical Immunology, National Institute of Tuberculosis and Lung Diseases; Warsaw; Poland

The editors of the Journal of Mother and Child/Medycyna Wieku Rozwojowego invite you to read our latest quarterly issue, the last in 2021. The topics discussed herein encompass antenatal treatment of respiratory distress syndrome and breastfeeding—also in premature infants—as well as maternal ultraprocessed food intake and child labour manipulation.

We start reading with a secondary observational analysis of the Lifestyle Intervention for Two (LIFT) randomized controlled trial data. Since there is a paucity of data related to mechanisms of health effects and dietary intake of ultraprocessed foods (UPF), the authors of this article aim to determine an association between maternal UPF intake and gestational weight gain (GWG) and neonatal body composition.

We continue with pregnancy-related studies, referring specifically to prematurity. We can read the results and authors’ conclusions based on a bidirectional (226 prospective and 42 retrospectives) cohort study performed to compare the respiratory distress syndrome and neonatal death among preterm twins from 28 to 34 weeks born < 14 days (Group A, n=268) and after 14 days (Group B, n=268) of completion of a single course of antenatal steroids.

Then we present two interesting articles concentrating on breastfeeding. Raczyńska and colleagues have analyzed the advantages of side-lying position (SLP) and semielevated position (SEP) during bottle-feeding in premature infants. They provide evidence that SLP reduces the number of choking episodes during feeding and improves the proportion of milk consumed. Another group, Grzyb et al., present Polish women’s opinions and experiences regarding breastfeeding in public. Based on a one-time 11-question survey that depicts women facing criticism while breastfeeding in public, the authors find it necessary to create dedicated places for breastfeeding in public places.

The issue closes with an interesting study aimed at looking into the existing trends of child labour abuse in Addis Ababa and analysing the sociocultural barriers that impede nongovernmental organizations. The key message from the findings of this study is that the Winrock International (2008) approach of CIRCLE experience with an awareness-raising plan is recommended.

We hope that the presented papers meet your expectations and encourage you to read other issues of our journal.

